# Novel Clofarabine-Based Combinations with Polyphenols Epigenetically Reactivate Retinoic Acid Receptor Beta, Inhibit Cell Growth, and Induce Apoptosis of Breast Cancer Cells

**DOI:** 10.3390/ijms19123970

**Published:** 2018-12-10

**Authors:** Katarzyna Lubecka, Agnieszka Kaufman-Szymczyk, Barbara Cebula-Obrzut, Piotr Smolewski, Janusz Szemraj, Krystyna Fabianowska-Majewska

**Affiliations:** 1 Department of Biomedical Chemistry, Medical University of Lodz, 92-215 Lodz, Poland; agnieszka.kaufman-szymczyk@umed.lodz.pl; 2 Department of Experimental Hematology, Medical University of Lodz, 93-510 Lodz, Poland; barbara.cebula@umed.lodz.pl (B.C.-O.); piotr.smolewski@umed.lodz.pl (P.S.); 3 Department of Medical Biochemistry, Medical University of Lodz, 92-215 Lodz, Poland; janusz.szemraj@umed.lodz.pl; 4 Faculty of Medicine, Lazarski University, 02-662 Warsaw, Poland; krystyna.fabianowska-majewska@lazarski.pl

**Keywords:** DNA methylation, breast cancer, retinoic acid receptor beta, clofarabine, EGCG, genistein

## Abstract

An epigenetic component, especially aberrant DNA methylation pattern, has been shown to be frequently involved in sporadic breast cancer development. A growing body of literature demonstrates that combination of agents, i.e. nucleoside analogues with dietary phytochemicals, may provide enhanced therapeutic effects in epigenetic reprogramming of cancer cells. Clofarabine (2-chloro-2′-fluoro-2′-deoxyarabinosyladenine, ClF), a second-generation 2′-deoxyadenosine analogue, has numerous anti-cancer effects, including potential capacity to regulate epigenetic processes. Our present study is the first to investigate the combinatorial effects of ClF (used at IC_50_ concentration) with epigallocatechin-3-gallate (EGCG, tea catechin) or genistein (soy phytoestrogen), at physiological concentrations, on breast cancer cell growth, apoptosis, and epigenetic regulation of retinoic acid receptor beta (*RARB*) transcriptional activity. In MCF7 and MDA-MB-231 cells, *RARB* promoter methylation and expression of *RARB*, modifiers of DNA methylation reaction (*DNMT1*, *CDKN1A*, *TP53*), and potential regulator of *RARB* transcription, *PTEN*, were estimated using methylation-sensitive restriction analysis (MSRA) and quantitative real-time polymerase chain reaction (qPCR), respectively. The combinatorial exposures synergistically or additively inhibited the growth and induced apoptosis of breast cancer cells, followed by *RARB* hypomethylation with concomitant multiple increase in *RARB*, *PTEN*, and *CDKN1A* transcript levels. Taken together, our results demonstrate the ability of ClF-based combinations with polyphenols to promote cancer cell death and reactivate DNA methylation-silenced tumor suppressor genes in breast cancer cells with different invasive potential.

## 1. Introduction

Breast cancer is a heterogeneous disease that constitutes the second most prevalent cancer in the world and major epidemiological problem [1]. Due to the increasing incidence and death rates of breast tumors among women worldwide, there is a desperate call for development of new effective strategies of early detection, prevention, and therapy. Over 30% of sporadic breast cancer cases have been shown to result from a variety of modifiable environmental and social factors, including sedentary lifestyle, poor dietary habits, obesity, cigarette smoking, and exogenous hormones. It implies that dysregulated epigenetic code seems to play a pivotal role in sporadic breast cancer development [2]. Epigenetic processes, which include mainly DNA methylation, covalent histone modifications, and regulation by non-coding RNAs, are involved in determining the transcriptional activity of certain genes. In cancerous cells, aberrant epigenetic marks may lead to silencing of tumor suppressor genes and concomitantly to overexpression of oncogenes and prometastatic genes [3,4,5,6,7]. Among variety of the mechanisms by which breast tumor growth occurs, there is one that is potentially reversible, DNA methylation-mediated silencing of tumor suppressor genes. The number of tumor suppressor genes have been shown to act as negative regulators of intracellular oncogenic signaling pathways [3,4,6,7,8,9,10,11]. Thus, discovering new complex strategies of epigenetic therapy to target and reactivate these genes in cancer cells seems really promising.

Nucleoside analogues constitute an important group among new anti-cancer epigenetic drugs. The synthetic nucleosides as antimetabolites in DNA and/or RNA synthesis have been successfully introduced not only into therapy of hematological malignancies and viral infections, but more often into treatment of solid tumors [3,4,6,8,9,10,11,12,13,14,15,16,17]. Epigenetic anti-cancer therapy focused on inhibiting DNA methylation processes includes different strategies. Specific inhibitors of DNA methyltransferases (DNMTs, the enzymes catalyzing DNA methylation reaction), such as azacitidine and decitabine (DAC), need to be mentioned. Upon incorporation into DNA, these cytidine analogues inhibit DNMT activity and cause hypomethylation and upregulation of certain tumor suppressor genes [8,9,12]. Alternative approach refers to compounds, such as cladribine (2-chloro-2′-deoxyadenosine, 2CdA), fludarabine (2-fluoroarabinosyladenine, F-ara-A), and clofarabine (2-chloro-2′-fluoro-2′-deoxyarabinosyladenine, ClF), which interfere with one carbon metabolism and inhibit the activity of *S*-adenosyl-l-homocysteine (SAH) hydrolase. The following changes in SAH and *S*-adenosyl-l-methionine (SAM, methyl group donor) levels, i.e., SAH accumulation and SAM pool depletion, cause disruption in active methyl cycle and subsequent inhibition of DNA methylation reaction [15,18,19,20,21].

Due to many limitations of epigenetic monotherapy, a growing number of anti-cancer nucleoside analogue-based combinatorial strategies focused on remodeling of aberrant DNA methylation patterns have been developed and are currently under investigation [4,6,10,11,16,17]. Our recent discoveries [4] support the enhanced chemotherapeutic potential of the combinations of dietary bioactive compounds with conventional drugs in epigenetic anti-cancer therapy [4,6,10,11]. We showed previously that ClF used in combination with sulforaphane (SFN, an isothiocyanate from broccoli, brussels sprouts, and cabbage), demonstrated potent growth inhibitory activity in human breast cancer cells and led to robust hypomethylation and upregulation of *RARB* (retinoic acid receptor beta) and *PTEN* (phosphatase and tensin homologue) tumor suppressor genes, especially in mildly malignant breast cancer cells [4]. These two tumor suppressor genes, DNA methylation-silenced in breast cancer [22,23,24,25,26] have been chosen to investigate the chemopreventive potential of tested ClF-based combinations with different bioactive phytochemicals. RARB is a tumor suppressor protein that modulates cell proliferation and differentiation, cell cycle progression, and apoptosis [27]. RARB can act as an effective suppressor of transcriptional activity of AP-1 (activator protein 1) protein complex [28,29]. *PTEN* encodes protein involved in downregulation of intracellular oncogenic signaling pathways, such as phosphoinositide 3-kinase (PI3K)/AKT and mitogen-activated protein kinase (MAPK)/AP-1 [30,31]. AP-1 is a transcription factor positively regulating *DNMT1* (DNA methyltransferase 1) gene encoding the main enzyme responsible for catalysis of DNA methylation reaction [31]. Thus, the proteins encoded by *RARB* and *PTEN*, that are negative regulators of AP-1, might be indirectly involved in *DNMT1* downregulation [32,33]. Moreover, Lefebvre and colleagues documented that *RARB* expression may be further induced by PTEN [34].

Numerous studies have been set to get a better understanding of novel epigenetic chemopreventive approaches with usage of dietary phytochemicals in cancer [4,6,10,11,35,36]. Certain bioactive polyphenols, especially when used at low doses that are within the range of physiological concentrations, have been shown to exert substantial anti-cancer effects through remodeling of the epigenetic marks rather than robust alterations in the epigenome, frequently observed for synthetic pharmacological agents such as DAC [4,6,7,10,11,12,35,36,37]. Therefore, in the present study, we investigated the effects of ClF in combination with well-known and widely studied polyphenols: Epigallocatechin gallate (EGCG, tea catechin) or genistein (soy phytoestrogen), potent inhibitors of DNA methyltransferases (DNMTs) and modulators of histone modifications [38], on *RARB* methylation and expression in well-defined in vitro model of human breast cancer cell lines with different invasive potential. MCF7 (mildly malignant, ER-positive, wild-type p53; functional deletion in the caspase 3 (*CASP3*) gene) and MDA-MB-231 (malignant, ER-negative, mutant p53) breast cancer cell lines were chosen as an in vitro experimental model of human solid tumors.

Moreover, in an effort to understand some of epigenetic mechanisms underlying any changes in *RARB* transcriptional activity upon the tested combinatorial exposures in breast cancer cells, we assessed expression levels of known DNA methylation modifiers, *DNMT1*, *CDKN1A* (*p21*), and *TP53*, as well as potential regulator of *RARB* transcription, *PTEN*. *TP53* is a tumor suppressor relevant for regulation of cellular growth, cell cycle and apoptosis. *TP53* gene encodes p53 protein that acts as a transcription factor for a numerous p53-inducible genes, i.a. positively affecting *CDKN1A* [39,40] and downregulating *DNMT1* [41]. It has been reported, that during DNA replication, p21 tumor suppressor encoded by *CDKN1A* competes with DNMT1 for the same binding site on proliferating cell nuclear antigen (PCNA, homotrimeric ring surrounding DNA), which disrupts DNMT1/PCNA complex formation and subsequently may cause inhibition of DNA methylation reaction [42,43].

The selected polyphenols, EGCG and genistein, have been shown to reverse DNA methylation-mediated silencing of tumor suppressor genes and inhibit growth and promote death of breast, cervical, esophageal, and/or prostate cancer cells [44,45]. The presence of catechol group in the structure of EGCG play a key role in inhibiting DNMT activity. EGCG is an excellent substrate for the methylation reaction mediated by cathecol-O-methyltransferase (COMT). Followed by COMT-mediated methylation reactions, SAM pool depletion and SAH formation have been observed, and SAH accumulation is a potent reverse inhibitor of DNA methylation [46]. Moreover, this tea constituent was demonstrated to directly interact with the catalytic site of DNMT1 [45]. The epigenetic activity of genistein, a potent phytoestrogen, can be attributed to their ability to stimulate *CDKN1A* via estrogen response elements (ERE) within its promoter [47], as well as to repress AP-1 transcriptional activity [48] or *PTEN* upregulation [49]. In 2014 Xie and colleagues, using molecular modeling, demonstrated that genistein may directly interact with the catalytic domain of DNMT1, and competitively inhibit the binding of hemimethylated DNA to this domain [50].

Our present study is the first to investigate the combinatorial effects of ClF (used at IC_50_ concentration) with polyphenols, EGCG, or genistein used at the range of physiological concentrations, on breast cancer cell growth, apoptosis, and epigenetic regulation of transcriptional activity of DNA methylation-silenced tumor suppressor genes, such as *RARB* and *PTEN*.

## 2. Results and Discussion

### 2.1. ClF-Based Combinations with Polyphenols Inhibits Breast Cancer Cell Growth

**Figure 1:**
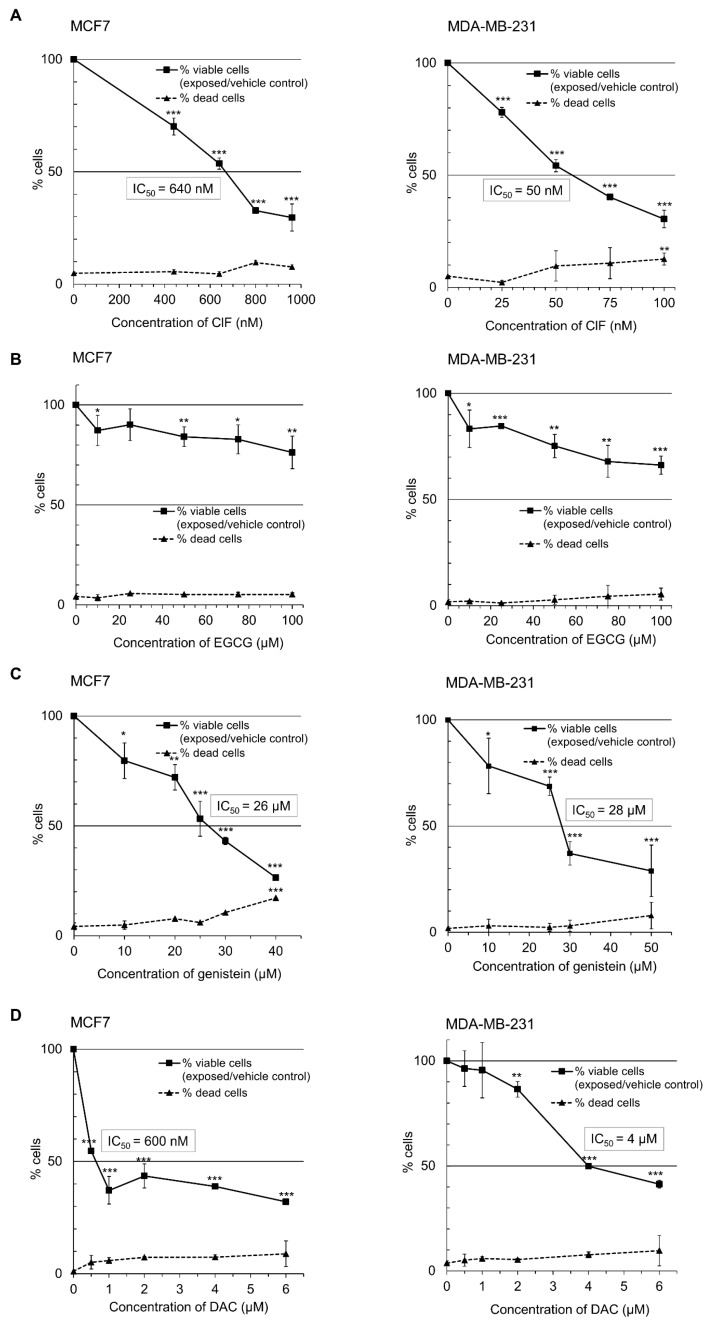
Effect of ClF (**A**), EGCG (**B**), genistein (**C**), and DAC (**D**) on MCF7 (left panel) and MDA-MB-231 (right panel) cell viability as measured by trypan blue exclusion test. MCF7 and MDA-MB-231 breast cancer cells were incubated for 4 days with the tested compounds at the indicated concentrations. The number of viable cells after 4 days exposure to ClF, genistein, and DAC at IC_50_ concentrations (IC_50_ not achieved for EGCG) was expressed as a percentage of viable cells in the vehicle control ((viable exposed/viable vehicle control)*100%). The number of dead cells in either vehicle control or exposed group was calculated as a percentage of the total cell number. DAC was used as a positive control in the trypan blue exclusion test. Values are means ± SD of at least three independent experiments. Exposure (ClF alone or EGCG alone or genistein alone or DAC alone) versus vehicle control, *** *p* < 0.001, ** *p* < 0.01, * *p* < 0.05.

Our findings demonstrate that all the tested compounds, ClF, EGCG, and genistein, decrease breast cancer cell growth with low toxicity. As measured by trypan blue exclusion test, they inhibit MCF7 and MDA-MB-231 cell viability in a dose-dependent manner (Figure 1).

Following 4 days-exposure, ClF concentrations leading to 50% decrease in the number of viable cells (IC_50_) were determined as 640 nM in non-invasive MCF7 cells and 50 nM in highly invasive MDA-MB-231 cells (Figure 1A) [3]. The number of dead cells did not exceed 10% regardless of cell invasiveness indicating low cytotoxicity level of ClF used at IC_50_ concentrations (Figure 1A). Noticeably, ClF reduced viability of malignant ER-negative MDA-MB-231 cells by 50% at over 10-fold lower concentration (IC_50_ = 50 nM) compared with mildly malignant ER-positive MCF7 cells (IC_50_ = 640 nM) [3] (Figure 1A). Varying ClF efficacy with respect to the level of cell growth inhibitory properties could be expected due to the varying characteristics of each cell line.

These observations may be associated with different p53 status. MCF7 cells express wild-type p53, while in MDA-MB-231 cells p53 is mutated [39]. However, in our previous and present studies no significant changes in *TP53* expression were observed upon ClF exposure in either of the cell lines [3]. Another potential explanation is that MCF7 cells possess a functional deletion of the caspase 3 (*CASP3*) gene [51].

Moreover, it has been demonstrated that responsiveness to the clinically important nucleoside analogues may be critically determined by deoxycytidine kinase (DCK) expression. This enzyme is required to convert the inactive prodrugs into their pharmacologically active forms [15,52]. In 2012, Geutjes and colleagues reported that DCK is expressed at higher levels in breast cancer patients that are at high risk of recurrence and having poor clinical outcome. The DCK overexpression in these patients was strongly correlated with a favorable response to nucleoside analogues [52]. In support of this, a causal relationship between DCK levels and sensitivity to the nucleoside analogues in breast cancer cell lines was reported as well [52].

Interestingly, in 2015 Abramczyk and colleagues showed the significant difference in the number of cytoplasmic lipid droplets in non-malignant (normal-like) MCF10A mammary epithelial cells and mildly malignant MCF7 or malignant MDA-MB-231 breast epithelial cells. They found around 2 and 4 times more cytoplasmic lipid droplets in MCF7 and MDA-MB-231, respectively, compared to MCF10A cells [53]. Together with lipophilic properties of ClF [15] and almost two-fold higher proliferation rate in MDA-MB-231 versus MCF7 cells [3], it may partly explain greater bioavailability of the drug in invasive MDA-MB-231 cells.

EGCG and genistein exerted similar effects towards non-invasive and invasive breast cancer cells (Figure 1B,C). Upon 4 days-exposure to EGCG at the concentration range to 100 µM, IC_50_ concentrations have not been achieved regardless of cell invasiveness (Figure 1B). Genistein administration led to 50% decrease in the number of viable cells (IC_50_) at the 26–28 µM concentrations both in non-invasive ER-positive MCF7 and highly invasive ER-negative MDA-MB-231 cells (Figure 1C).

It is important to notice that any variances between different studies investigating the effects of the selected polyphenols on cellular viability of MCF7 and MDA-MB-231 cells may be associated with different cell culture conditions. For example, preparation of stock solutions of the tested compounds with usage of different type of solvent and its final concentration, i.e., water or DMSO for EGCG, and DMSO or ethanol for genistein. Moreover, different than recommended culture media, i.e., RPMI-1640 or DMEM medium instead of EMEM or L-15 media for MCF7 and MDA-MB-231 cells, respectively. Different schedules and durations of cell exposure to the mentioned compounds may affect the extent of their anti-proliferative activity as well [50,54,55,56].

Combination of EGCG with ClF significantly enhanced inhibition of cell growth in both MCF7 and MDA-MB-231 cells as compared with ClF alone (Table 1A,B), according to CompuSyn software analysis [57]. This synergistic effect was achieved at only 10 µM concentration of EGCG that reflects physiologically relevant EGCG levels in humans (Figure 1B; Table 1A,B) [38]. Additive decrease in the number of viable cells was also observed in both cell lines after combinatorial ClF and genistein (10 µM) exposure compared with ClF alone (Table 1A,B).

### 2.2. Both EGCG and Genistein Enhances ClF Pro-Apoptotic Effects Mostly in Mildly Malignant MCF7 Cells

**Figure 2:**
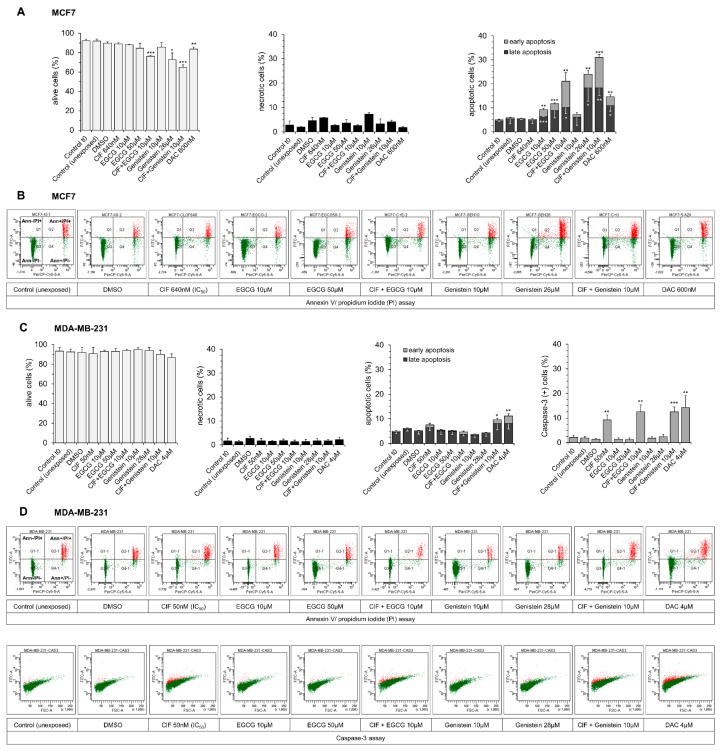
Effects of ClF (IC_50_) and EGCG (10 µM) or genistein (10 µM) alone and in combination on the number of alive, apoptotic and necrotic cells as well as on caspase 3 activity (only in MDA-MB-231 cells) in MCF7 (**A**,**B**) and MDA-MB-231 (**C**,**D**) cell lines. The original FACS cytograms of annexin V/propidium iodide and caspase 3 (only in MDA-MB-231 cells) assays for MCF7 (**B**) and MDA-MB-231 (**D**) cells are shown. Values are means ± S.D. of three independent experiments. Exposure versus vehicle control: *** *p* < 0.001, ** *p* < 0.01, * *p* < 0.05. Ct0, control cells used for experiments (time 0 h); Control (unexposed), negative control, cells incubated with medium; DAC, positive control; DMSO, vehicle control; Q1, necrotic cells (Ann−/PI+); Q2, late apoptotic cells (Ann+/PI+); Q3, viable cells (Ann −/PI−); Q4, early apoptotic cells (Ann+/PI−).

A dose-dependent increase in the number of apoptotic MCF7 cells from 5% in the vehicle control to 9% and 12% in the samples incubated with EGCG used alone at 10 µM and 50 µM concentrations, respectively, was determined upon 4 days-exposure (Figure 2A,B). This observation was not associated with caspase 3 activation (data not shown), which is consistent with Jänicke’s report showing lack of functional caspase 3 in MCF7 cells [51]. It may indicate that the tested polyphenol induces apoptosis through caspase-independent pathway in this cell line. Huang and colleagues demonstrated that EGCG-induced apoptosis of MCF7 cells may be associated with Bcl-2 downregulation [55].

In invasive MDA-MB-231 cells that express caspase 3, EGCG did not increase either apoptotic cell rate (Figure 2C,D) or caspase 3 activity (Figure 2C,D), indicating no effect on caspase-dependent or caspase-independent apoptotic pathway. Moreover, lack of functionally active p53 in MDA-MB-231 cells may account for their resistance to EGCG pro-apoptotic effects (Figure 2C,D). Hong and colleagues observed that EGCG inhibits the growth and induces apoptosis of malignant MDA-MB-231 cells through inactivation of β-catenin signaling pathway, but at much higher dose of EGCG equal to 200 µM [56].

Upon 4 days-exposure to genistein used alone, we observed more profound dose-dependent increase in the number of apoptotic MCF7 cells, from 5% in the vehicle control to 7% and 24% in the samples incubated with 10 µM and 26 µM concentrations, respectively (Figure 2A,B).

Similarly to EGCG, genistein did not increase either apoptotic cell rate or caspase 3 activity in MDA-MB-231 cells (Figure 2C,D). These results are consistent with the report of Xu and Loo, showing that even after 6 days of incubation with 50 μM genistein, only MCF7, but not MDA-MB-231 cells, showed morphological signs of apoptosis [58].

In our previous study, we found 10 µM SFN in combination with ClF to work well together at increasing apoptosis in mildly malignant MCF7 cells [4]. Therefore, we chose to assess pro-apoptotic effects of novel ClF-based combinations with selected polyphenols, EGCG, and genistein, at the same 10 µM concentration, that is at the range of their physiologically relevant levels in humans [38].

In MCF7 cells, both EGCG (10 µM) and genistein (10 µM) significantly enhanced pro-apoptotic effects of ClF (IC_50_) after 4 days-exposure. The number of apoptotic cells increases from 5% after ClF alone to 21% and 31% after combinatorial ClF + EGCG and ClF + Genistein administration, respectively (Figure 2A,B).

In MDA-MB-231 cells, EGCG in combination with ClF did not change significantly ClF effects on cell death (Figure 2C,D). Although, ClF in combination with genistein caused slight increase in the number of apoptotic MDA-MB-231 cells, from 7% after ClF alone to 10% upon combinatorial exposure (Figure 2C,D). Interestingly, upon 4 days-incubation with the tested combinations, almost 13% of all MDA-MB-231 cells showed active caspase 3, whereas after ClF alone approximately 9% of all MDA-MB-231 bound antibodies against caspase 3 (Figure 2C,D). It may indicate the initiation of early stage of caspase-dependent apoptotic pathway in invasive MDA-MB-231 cells (Figure 2C,D).

In both cell lines, combinatorial ClF and EGCG or genistein did not cause cell necrosis as measured by flow cytometry (Figure 2).

DAC used at IC_50_ concentrations caused significant induction of apoptosis in both MCF7 (about 15% of apoptotic cells) and MDA-MB-231 (over 11% of apoptotic cells) cells (Figure 2). Upon DAC exposure, almost 14% of all MDA-MB-231 cells showed active caspase 3 (Figure 2C,D), which might suggest caspase-dependent induction of apoptosis in this highly malignant breast cancer cell line. In both cell lines, DAC did not cause a relevant increase in cell necrosis rate (Figure 2).

### 2.3. ClF, EGCG, and Genistein Used Alone and in Combinations Exert Profound Effects on RARB Promoter Methylation and Expression in Breast Cancer Cells

We showed recently that ClF used alone and in combination with SFN demonstrated potent growth inhibitory activity in human breast cancer cells and led to robust hypomethylation and upregulation of DNA methylation-silenced *RARB* tumor suppressor gene with concomitant *DNMT1* downregulation and *CDKN1A* upregulation, especially at early stage of breast cancer development [4]. Therefore, we decided to examine the effect of novel ClF-based combinations with polyphenols on *RARB* transcriptional activity and determine if there are any alterations in DNA methylation levels within its proximal promoter region. In an effort to understand some of the mechanisms underlying our observations we next assessed exposure-mediated changes in the expression of known epigenetic modifiers, *DNMT1*, *CDKN1A*, and *TP53*.

**Figure 3:**
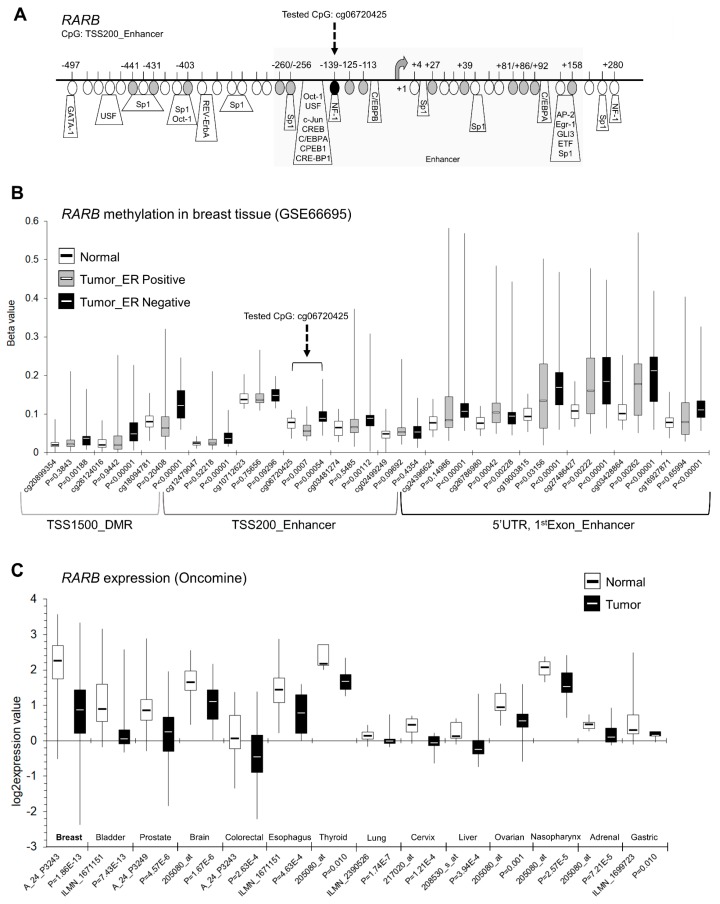
Relevance of DNA methylation-mediated silencing of *RARB* tumor suppressor in human breast cancer in vivo. (**A**) A map of the *RARB* proximal promoter region (Human GRCh37/hg19 Assembly) including an enhancer region. The methylation-sensitive restriction analysis (MSRA)-tested CpG site (located within the enhancer region of the *RARB* promoter, -139 bp from transcription start site (TSS); cg06720425 on Illumina 450K microarray platform; chr3: 25469694) is depicted as a black circle, indicated with black dashed arrow. The neighboring CpG sites [-441 bp to +158 bp from TSS] covered on Illumina 450K array are depicted as gray circles. Putative transcription factor binding sites are indicated as predicted using TransFac. (**B**) Methylation status of these 14 CpG sites (black and gray circles on the gene map) within the *RARB* proximal promoter region, covered on Illumina 450K array and expressed as beta value in breast tumors based on publicly available Illumina 450K data (GSE66695). The tested CpG site, cg06720425, is indicated with black dashed arrow. Beta value, the methylation score for a specific CpG site with any values between 0 (unmethylated) and 1 (completely methylated), according to the fluorescent intensity ratio. (**C**) Breast cancer gene expression microarray data for *RARB* from Oncomine. The normal versus tumor gene expression data are presented as log2-transformed median centered per array, and SD-normalized to 1 per array. The demonstrated changes are statistically significant (*P* < 0.05).

According to publicly available Illumina 450K data (GSE66695), the *RARB* proximal promoter region is shown to be hypermethylated both in ER-positive and ER-negative breast tumors as compared with normal tissue (Figure 3B). The higher extent of *RARB* hypermethylation was observed in ER-negative tumors (Figure 3B). Moreover, Oncomine data indicate that *RARB* is transcriptionally silenced in many types of cancers, with breast cancer at the forefront (Figure 3C). The detailed map in Figure 3A demonstrates the exact position of the tested CpG site, cg06720425 (marked in black) within *RARB* enhancer of its promoter region (−139 bp from transcription start site, TSS) and 13 neighboring CpGs covered on Illumina 450K microarray platform (marked in gray, from −441 bp to +158 bp in relation to TSS) (Figure 3A). Putative transcription factor binding sites are depicted on the gene map, as predicted using TransFac (Figure 3A). The number of binding sites for DNA methylation-sensitive transcription factors within the tested *RARB* promoter fragment enhanced its potential regulatory role in *RARB* transcription (Figure 3A) [22,27].

**Figure 4:**
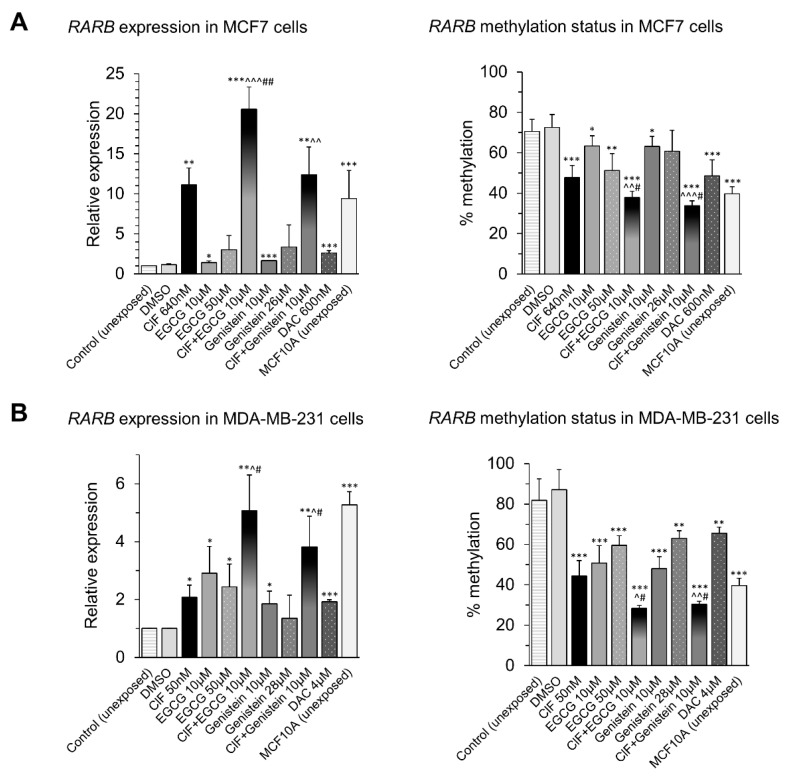
Effects of ClF (IC_50_) and EGCG (10 µM) or genistein (10 µM) alone and in combination on expression (left panel) and methylation (right panel) of *RARB* tumor suppressor gene in MCF7 (**A**) and MDA-MB-231 (**B**) cells. All results represent mean ± SD of three independent experiments. Exposure (ClF alone, EGCG alone, genistein alone, ClF + EGCG or ClF + Genistein) versus vehicle control, *** *p* < 0.001, ** *p* < 0.01, * *p* < 0.05. EGCG or genistein alone versus combinatorial exposure (ClF + EGCG or ClF + Genistein), ^^^ *p* < 0.001, ^^ *p* < 0.01, ^ *p* < 0.05. ClF alone versus combinatorial exposure (ClF + EGCG or ClF + Genistein), ## *p* < 0.01, # *p* < 0.05.

Both EGCG and genistein as the potent DNMTs inhibitors can act as effective modulators of DNA methylation processes in cancer cells [38]. Fang and colleagues, as well as Khan and colleagues, observed EGCG-mediated hypomethylation of *RARB* promoter and its upregulation in human cervical, esophageal, colon, and prostate cancers [59,60]. According to Fang’s and Sundaram’s reports, genistein induced DNA methylation-mediated reactivation of *RARB* both in esophageal, prostate, and cervical cancers [44,61]. In our current studies, we identified a role of EGCG and genistein in regulation of promoter methylation and expression of *RARB* tumor suppressor gene in human breast cancer cells with different invasive potential (Figure 4).

Four days-exposure of MCF7 cells to the tested compounds used alone, ClF, EGCG, and genistein, led to significant increases in *RARB* mRNA levels, the highest upon ClF (11-fold increase) (Figure 4A, left panel). The dose-dependent *RARB* re-expression (from 1.4-fold to 3.4-fold increases in *RARB* mRNA levels) after EGCG (10 and 50 µM) and genistein (10 and 26 µM) administration were observed in MCF7 cells (Figure 4A, left panel). *RARB* transcriptional reactivation was accompanied by significant decrease in *RARB* promoter methylation by approximately 10–25%. Again, the highest changes were observed followed by MCF7 cells incubation with ClF (Figure 4A, right panel). *RARB* promoter methylation was attenuated by around 10–22% at both concentrations of EGCG and genistein (Figure 4A, right panel). These observations in MCF7 cells (Figure 4A) indicate that the tested compounds may regulate *RARB* transcription at least partly through its promoter methylation at non-invasive stage of breast cancer. Noticeably, DAC (600 nM) used in our present study as a reference agent, acting as a potent DNMTs inhibitor, led to similar decrease in *RARB* promoter methylation as ClF (by 24–25% after ClF or DAC), but it was associated with only 2.6-fold increase in *RARB* expression comparing to 11-fold increase upon ClF exposure (Figure 4A).

In invasive MDA-MB-231 cells, the tested compounds used alone caused relevant *RARB* upregulation, at the range of 1.4–2.9-fold changes in *RARB* transcript levels. The strongest alterations were observed upon 10 µM EGCG exposure (2.9-fold increase compared to 2.1-fold increase after ClF) (Figure 4B, left panel). The effects on *RARB* transcriptional activity and its promoter methylation did not show a dose-dependent pattern. Both EGCG and genistein at 10 µM concentration attenuated *RARB* promoter methylation by 36% and 39% that was associated with 2.9- and 1.9-fold increase in gene expression, respectively (Figure 4B). However, 50 µM EGCG and 28 µM genistein reduced *RARB* promoter methylation by 28% and 24%, increasing gene expression by only 140% (2.4-fold increase) and 40%, respectively (Figure 4B). 4 days-exposure of MDA-MB-231 cells to 4 µM DAC led to the lowest decrease in *RARB* promoter methylation compared to other tested compounds. The methylation state of *RARB* promoter changed from 73% in vehicle control to 66% after DAC exposure, elevating *RARB* mRNA level by 90% (1.9-fold increase) (Figure 4B).

The extent of hypomethylation induced by the tested compounds used alone was more robust in MDA-MB-231 cells where basal level of *RARB* promoter methylation in unexposed cells is higher compared with MCF7 cells. *RARB* promoter fragment is methylated at 70% in MCF7 and at 82% in MDA-MB-231 cells (Figure 4A,B, right panels). Moreover, taking into account that DNA methylation is a postreplicative modification, almost 2-fold higher proliferation rate of MDA-MB-231 versus MCF7 cells may account for stronger manifestation of methylation alterations caused by the tested chemicals in this cell line [3].

In MCF7 cells, both ClF-based combinations with EGCG and genistein enhances the inhibitory effect of ClF on *RARB* promoter methylation after 4 days-exposure (Figure 4A, right panel). The methylation level drops from 48% after ClF alone (73% in vehicle control) to 38% and 34% after ClF + EGCG and ClF + Genistein combinations, respectively (Figure 4A, right panel). These changes in methylation marks correlate with stronger induction of *RARB* expression, especially after combinatorial ClF and EGCG administration (Figure 4A, left panel). Interestingly, this combined exposure leads to over 20-fold increase in *RARB* mRNA level which is highly relevant compared to 11-fold overexpression after ClF alone (Figure 4A, left panel). This robust effect on *RARB* expression does not coincide with enhancement of hypomethylation level within *RARB* promoter (Figure 4). It suggests the involvement of another mechanisms in the combined effects of ClF and EGCG on *RARB* transcription.

Similarly to mildly malignant MCF7 cells, in highly malignant MDA-MB-231 cells, both EGCG and genistein in combination with ClF cause additional changes in both *RARB* promoter methylation and gene expression (Figure 4B). The methylation level decreases from 44% after ClF alone (87% in vehicle control) to 28% and 30% after ClF + EGCG and ClF + Genistein combinations, respectively (Figure 4B, right panel). These changes in promoter methylation marks are associated with enhanced induction of *RARB* expression, but to a lesser extent than in MCF7 cells (Figure 4B, left panel). In MDA-MB-231 cells, the combined exposures, ClF + EGCG and ClF + Genistein, lead to 5-fold and almost 4-fold increases in *RARB* mRNA level, respectively, which are around 3–4 times lower compared to over 20-fold or 12-fold overexpression after combinatorial administrations in MCF7 cells (Figure 4A,B, left panels). The more robust *RARB* upregulation induced by the tested ClF-based combinations with polyphenols in MCF7 cells compared to MDA-MB-231 cells, may be explained by 2 times lower basal level of *RARB* transcript in unexposed MCF7 cells than in unexposed MDA-MB-231 cells, as compared with unexposed non-malignant (normal-like) MCF10A cells (Figure 4A,B, left panels). It may also indicate some distinct mechanisms of the combined exposures in non-invasive compared to invasive breast cancer cells.

It is worth pointing out that the observed *RARB* methylation drops and expression rises, followed by the tested combinatorial exposures both in MCF7 and MDA-MB-231 breast cancer cells, alter “cancer-specific” *RARB* methylation and expression states towards “normal-like” *RARB* methylation and expression levels determined in unexposed non-malignant mammary epithelial MCF10A cells (Figure 4).

### 2.4. RARB Transcriptional Reactivation in Response to the ClF Combinations with Polyphenols Is Associated with Robust CDKN1A Upregulation

**Figure 5:**
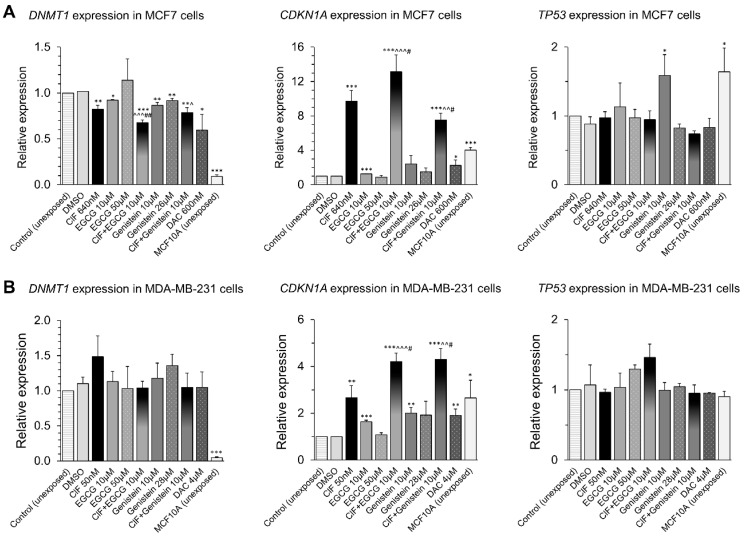
Effects of ClF (IC_50_) and EGCG (10 µM) or genistein (10 µM) alone and in combination on expression of modifiers of DNA methylation reaction, *DNMT1*, *CDKN1A*, and *TP53*, in MCF7 (**A**) and MDA-MB-231 (**B**) cells. All results represent mean ± SD of three independent experiments. Exposure (ClF alone, EGCG alone, genistein alone, ClF + EGCG or ClF + Genistein) versus vehicle control, *** *p* < 0.001, ** *p* < 0.01, * *p* < 0.05. EGCG or genistein alone versus combinatorial exposure (ClF + EGCG or ClF + Genistein), ^^^ *p* < 0.001, ^^ *p* < 0.01, ^ *p* < 0.05. ClF alone versus combinatorial exposure (ClF + EGCG or ClF + Genistein), ## *p* < 0.01, # *p* < 0.05.

In an effort to gain better understanding of the epigenetic mechanisms involved in the changes in *RARB* transcriptional activity induced by the ClF-based combinations with the tested polyphenols, we determined changes in the expression of known epigenetic modifiers, *DNMT1*, *CDKN1A*, and *TP53* (Figure 5).

In MCF7 cells, both EGCG and genistein used alone at 10 µM concentration significantly increased *CDKN1A* expression by 1.3-fold and 2.4-fold, respectively, upon 4 days-exposure (Figure 5A, middle panel). As p53 protein encoded by *TP53* tumor suppressor gene was shown to act as a transcription factor regulating *CDKN1A* expression [62], we also examined the effect of the tested polyphenols on *TP53* expression (Figure 5, right panel). Increase in *TP53* mRNA levels by almost 60% was observed only at 10 µM genistein concentration, but not at higher concentration of this polyphenol (Figure 5A, right panel). Interestingly, the higher polyphenol concentration, the lower *CDKN1A* induction or no significant changes were observed, which corresponded with *TP53* expression. This suggests that 4 days-exposure to low dose (10 µM) genistein regulates *CDKN1A* transcription through p53-dependent pathway in MCF7 cells [62], and that ClF and EGCG at the tested concentration range may involve p53-independent pathway in these cells [15,55].

As both EGCG and genistein, potent DNMTs inhibitors, led to changes in DNA methylation and expression of *RARB* tumor suppressor gene with concomitant *CDKN1A* upregulation in MCF7 cells, we expected to detect significant changes in *DNMT1* expression (Figure 5A, left panel). We observed that 4 days-exposure to EGCG and genistein used alone cause significant 10% and 13% decrease in *DNMT1* transcript level mostly at 10 µM concentration, which is consistent with changes in *CDKN1A* expression upon these exposures (Figure 5A). ClF and DAC used alone led to significant 20% and 40% *DNMT1* downregulation in MCF7 cells, although not reaching the *DNMT1* expression level determined in unexposed non-malignant (normal-like) MCF10A cells (Figure 5A).

When MCF7 cells were exposed to ClF in combination with EGCG, the effect on *CDKN1A* expression increased as compared to ClF alone, from 9.7-fold change after ClF alone to 13.1-fold change upon ClF + EGCG exposure, that was associated with enhanced *DNMT1* downregulation by another 14% compared to ClF alone (Figure 5A, middle panel). Surprisingly, despite of relevant 10 µM genistein-mediated changes in *CDKN1A* and *TP53* expression, 20% reduction in *DNMT1* transcript level detected in ClF exposed cells was maintained and not enhanced upon the combinatorial administration (Figure 5A).

In MDA-MB-231 cells that express mutant p53, both EGCG and genistein used alone, only at 10 µM concentrations significantly increased *CDKN1A* expression by 60% and 100%, respectively (Figure 5B, middle panel), suggesting regulation of p53-independent pathway. Similarly to MCF7 cells, *CDKN1A* up-regulation was less profound when the polyphenols were used at higher concentrations (50 µM EGCG and 28 µM genistein) (Figure 5A, middle panel). Concomitantly, no suppression of *DNMT1* transcription was observed with a slight tendency for mRNA to increase at higher genistein dose as well as ClF used alone (Figure 5B, left panel).

The combinations of ClF and polyphenols in MDA-MB-231 cells resulted in over 4-fold induction of *CDKN1A* whereas exposure to ClF alone caused only 2.7-fold upregulation (Figure 5B, middle panel). It strongly suggests that the tested polyphenols and ClF are involved in regulation of *CDKN1A* through p53-independent pathway, presumably affecting the same elements of the pathway. As no significant alterations in *DNMT1* expression has been observed upon combinatorial exposures in MDA-MB-231 cells, it may suggest the involvement of another regulatory mechanisms of DNA methylation reaction.

**Figure 6:**
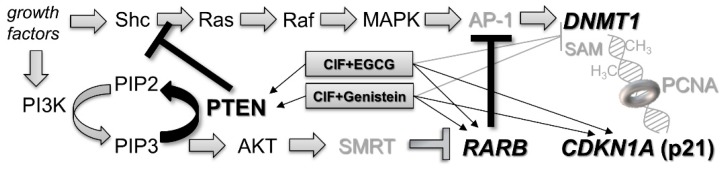
The potential repressive effects of the tested combinatorial exposures of ClF with EGCG or genistein on modulation of *DNMT1* transcription and/or activity in breast cancer cells. Implications of RARB and PTEN-mediated negative regulation of intracellular oncogenic signaling pathways, including MAPK/AP-1 and PI3K/AKT. A competition of CDKN1A (p21) with DNMT1 for the same binding site on PCNA. Shc, SH2-containing collagen-related proteins; MAPK, mitogen-activated protein kinase; AP-1, activator protein 1; PI3K, phosphatidylinositol-4,5-bisphosphate 3-kinase; PIP2, phosphatidylinositol (4,5)-bisphosphate; PIP3, phosphatidylinositol (3,4,5)-trisphosphate; SMRT, thyroid-, retinoic-acid-receptor-associated corepressor; SAM, *S*-adenosyl-l-methionine; PCNA, proliferating cell nuclear antigen.

Noticeably, the *CDKN1A* expression rises observed upon combinatorial exposures both in MCF7 and MDA-MB-231 cells, not only altered “cancer-specific” *CDKN1A* expression states towards “normal-like” *CDKN1A* expression levels determined in MCF10A cells, but even 2–3 times exceeded the “normal-like” *CDKN1A* expression pattern (Figure 4). As *CDKN1A* gene encodes p21 protein that competes with DNMT1 for the same binding site on PCNA during DNA replication, it can disturb in DNMT1/PCNA complex formation and affect DNMT1 activity and the rate of DNA methylation reaction [42,43] (Figure 6).

### 2.5. PTEN Upregulation upon Combinatorial Exposures Is Partly Involved in Robust RARB Transcriptional Reactivation

As in both MCF7 and MDA-MB-231, the robust effect on *RARB* expression upon combinatorial ClF + EGCG and ClF + Genistein exposures does not coincide with enhancement of hypomethylation level within *RARB* promoter (Figure 4), it may suggest the involvement of another mechanisms in the combinatorial effects on *RARB* transcription.

**Figure 7:**
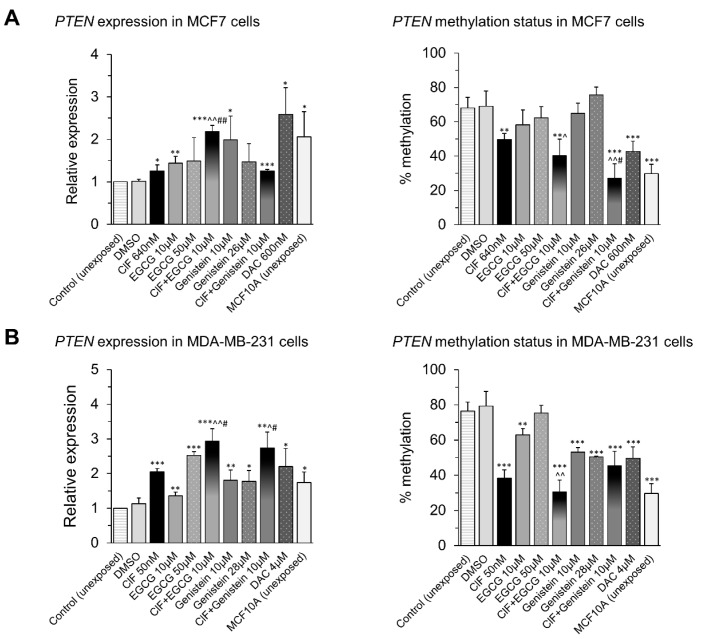
Effects of ClF (IC_50_) and EGCG (10 µM) or genistein (10 µM) alone and in combination on expression (left panel) and methylation (right panel) of *PTEN* tumor suppressor gene in MCF7 (**A**) and MDA-MB-231 (**B**) cells. All results represent mean ± SD of three independent experiments. Exposure (ClF alone, EGCG alone, genistein alone, ClF + EGCG or ClF + Genistein) versus vehicle control, *** *p* < 0.001, ** *p* < 0.01, * *p* < 0.05. EGCG or genistein alone versus combinatorial exposure (ClF + EGCG or ClF + Genistein), ^^ *p* < 0.01, ^ *p* < 0.05. ClF alone versus combinatorial exposure (ClF + EGCG or ClF + Genistein), ## *p* < 0.01, # *p* < 0.05.

One of the possible mechanisms is downregulation of PI3K/AKT signaling pathway by PTEN. PTEN, a dual specificity phosphatidyl inositol triphosphate (PIP3) phosphatase, antagonizes AKT signaling, causing diminished AKT-mediated phosphorylation and activity of its downstream effector, corepressor mediator for retinoic and thyroid hormone receptors (SMRT). It blocks recruitment of SMRT to *RARB* promoter, increases histone H3 and H4 acetylation, and finally stimulates *RARB* expression [34] (Figure 6). Therefore, we examined mRNA and promoter methylation levels of *PTEN* after 4 days-incubation with the tested compounds used alone and in combinations in MCF7 and MDA-MB-231 cells (Figure 7).

Following 4 days-exposure to ClF-based combinations with polyphenols, ClF + EGCG or ClF + Genistein, in both MCF7 and MDA-MB-231 cells, we observed significant (enhanced compared to ClF used alone) *PTEN* upregulation that was partly associated with concomitant *PTEN* hypomethylation within the CpG island of its proximal promoter region (CpG site, -149 bp from TSS) [24,25], especially in the case of ClF combination with EGCG (Figure 7). The extent of changes in *PTEN* methylation and expression levels upon combinatorial exposures was more robust than after DAC used alone in both tested breast cancer cell lines (Figure 7). Moreover, similarly to *RARB* gene, the observed *PTEN* methylation drops and expression rises, followed by combinatorial exposures both in MCF7 and MDA-MB-231 breast cancer cells, alter “cancer-specific” *PTEN* methylation and expression states towards “normal-like” *PTEN* methylation and expression levels determined in unexposed non-malignant MCF10A cells (Figure 7). These observations indicate that enhanced *PTEN* reactivation may be partly responsible for robust *RARB* upregulation in breast cancer cells upon ClF-based combinations with EGCG or genistein (Figure 4 and Figure 7).

## 3. Conclusions

Upon 4 days of exposure, the combinations of ClF with EGCG or genistein caused synergistic or additive inhibition of cell growth and greater induction of apoptosis, followed by significant *RARB* hypomethylation with concomitant multiple increase in *RARB*, *PTEN*, and *CDKN1A* transcript levels, mostly in mildly malignant MCF7 breast cancer cells. Moreover, in these cells, the aforementioned observations were associated with significant *DNMT1* downregulation. Our results support the role of combinatorial ClF and EGCG or genistein exposures in regulation of transcriptional activity of DNA methylation-silenced tumor suppressor genes, such as *RARB* and *PTEN*, which seem instrumental in breast cancer development.

Our current findings provide a relevant support behind the rationale to study ClF-based combinations with dietary polyphenols, such as EGCG and genistein, in more depth with regard to specific epigenetic mechanisms, including DNA methylation. Taken together, our results demonstrate the ability of ClF-based combinations with polyphenols to promote cancer cell death and reactivate DNA methylation-silenced tumor suppressor genes in human breast cancer cells with different invasive potential. We believe that further studies of combinatorial ClF and EGCG or genistein exposures may have translational significance through their potent chemopreventive activity against breast cancer.

## 4. Materials and Methods

### 4.1. Compounds

The tested nucleoside analogues, ClF and DAC, were purchased from Sigma-Aldrich (Poznan, Poland) and dissolved in water at the concentration of 1 mM. EGCG and genistein (Sigma-Aldrich, Poznan, Poland) were prepared in DMSO (Sigma-Aldrich, Poznan, Poland) (10 mM). All the solutions were stored at −20 °C.

### 4.2. Cell Culture

Human breast cancer cells, mildly malignant ER-positive MCF7 (ATCC HTB-22; tissue: Mammary gland, breast; derived from metastatic site: Pleural effusion; cell type: Epithelial; p53 wild-type; functional deletion in the caspase 3 (*CASP3*) gene) [51] and malignant ER-negative MDA-MB-231 (ECACC 92020424; tissue: Mammary gland, breast; derived from metastatic site: Pleural effusion; cell type: Epithelial; p53 mutant) were cultured according to American Type Culture Collection (ATCC, LGC Standards) and European Collection of Authenticated Cell Cultures (ECACC, Salisbury, UK) recommendations, respectively [3,4]. Non-malignant (normal-like) MCF10A cells (ATCC CRL-10317; tissue: Mammary gland, breast; cell type: Epithelial) were purchased in ATCC and cultured in basal medium, Mammary Epithelial Cell Growth Medium (MEGM) from Lonza AG (Warsaw, Poland) supplemented with additives from BulletKit (Lonza AG): hEGF (human epidermal growth factor), BPE (bovine pituitary extract), hydrocortisone, and human recombinant insulin. According to ATCC protocol, the gentamycin-amphotericin B mix provided with the kit has not been used in MCF10A cell culture. To prepare the complete growth medium, we added 1 U/mL 1 penicillin, 1 μg/mL 1 streptomycin (Gibco, Scotland, UK) and 100 ng/mL cholera toxin (Sigma-Aldrich, Poznan, Poland), purchased separately.

MCF7 and MDA-MB-231 cells were cultured in EMEM medium (MEM Eagle with Earle’s BSS, Lonza AG) and L15 medium (Leibovitz’s L15 medium, Lonza AG), respectively, supplemented with: 2 mM L-glutamine; 0.01 mg/mL bovine insulin (only for MCF7 cells) (Sigma-Aldrich, Poznan, Poland); 10% (and 15% for MDA-MB-231 cells) fetal bovine serum (FBS); 1 U/mL penicillin, and 1 μg/mL streptomycin (Gibco, Scotland, UK).

Cells were grown at 37 °C in a humidified atmosphere of 5% CO_2_, except for MDA-MB-231 cells incubated without CO_2_. Cells were subcultured every 3–4 days after reaching 70–80% confluency. For the experiments, MCF7, MDA-MB-231, and MCF10A cells were seeded at a low density of 22 × 10^3^, 16 × 10^3^, and 10 × 10^3^/cm^3^, respectively. After 24 h, the tested compounds diluted in full fresh medium was added and the incubation of MCF7 and MDA-MB-231 was continued for 4 days. MCF10A human mammary epithelial cells were used as a non-malignant (normal-like) control.

**Figure 8:**
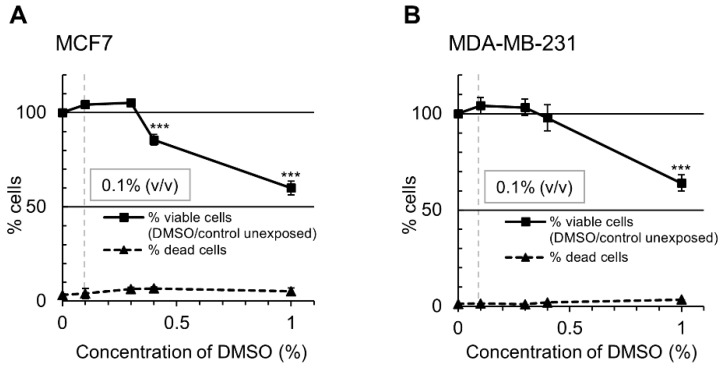
Effect of DMSO on MCF7 (**A**) and MDA-MB-231 (**B**) cell viability as measured by trypan blue exclusion test. MCF7 and MDA-MB-231 breast cancer cells were incubated for 4 days with DMSO at the indicated concentrations. The number of viable cells after 4 days exposure to DMSO was expressed as a percentage of viable cells in the unexposed control ((viable DMSO/viable unexposed control)*100%). The number of dead cells in either unexposed control or DMSO-exposed group was calculated as a percentage of the total cell number. DMSO at 0.1% (*v*/*v*) concentration was used as a vehicle control in all experiments. Values are means ± SD of at least three independent experiments. DMSO versus unexposed control, *** *p* < 0.001.

MCF7 and MDA-MB-231 cells were cultured for 4 days with ClF, EGCG, and genistein at different concentrations and a dose leading to 50% inhibition of cell growth (IC_50_) was established (not achieved for EGCG). EGCG or genistein at 10 µM concentration reflecting physiologically relevant levels was then combined with ClF, applied at previously determined IC_50_ concentrations (640 nM in MCF7 and 50 nM in MDA-MB-231 cells) [3]. DMSO was used as a vehicle control at 0.1% (*v*/*v*) concentration (Figure 8). After incubations genomic DNA or total RNA were extracted and purified.

The MCF7 and MDA-MB-231 cells were also incubated with 5-aza-2′-deoxycytidine (decitabine, DAC) at IC_50_ concentrations previously determined as equal to 0.6 µM and 4 µM [9] (Figure 1D). This deoxycytidine analogue, acting as a potent *DNMT1* inhibitor [12], was used in our studies as a reference agent (Figure 4, Figure 5 and Figure 7) and a positive control in cell viability and apoptosis assays [3,9] (Figure 1 and Figure 2).

### 4.3. Cell Viability and Apoptosis

Cell viability was measured by trypan blue (Sigma-Aldrich, Poznan, Poland) exclusion test and the cytostatic index (IC_50_) was determined. The number of viable cells after 4 days of exposure to CIF or EGCG or genistein or DAC was given as a percentage of viable cells in the vehicle control. The IC_50_ value represents the growth inhibitory concentration at which the compound causes 50% decrease in the number of viable cells compared with vehicle control after 4 days incubation. The number of dead cells that took up trypan blue was specified as the percentage of the total cell number. Cells were counted using Countess automated cell counter (Invitrogen, Life Technologies, Warsaw, Poland). The number of viable, necrotic, early, and late apoptotic cells was determined after 4 days compound exposure by flow cytometry analysis using annexin V/propidium iodide (PI) (FITC Annexin V Apoptosis Detection Kit, BD Pharmingen, Warsaw, Poland) staining according to the manufacturer’s protocol. Caspase-3 assay (Caspase-3 Assay Kit, BD Pharmingen, Warsaw, Poland) was performed to estimate its activity as a marker of the early stage of caspase-dependent apoptotic pathway in MDA-MB-231 cells (in MCF7 cells functional deletion in *CASP3* gene) [51].

### 4.4. CompuSyn

The available online CompuSyn software (http://www.combosyn.com/) was used to determine the combinatorial effect of ClF and EGCG or genistein. A combination index (CI) value greater than 1 indicates antagonism, a value below 1 denotes synergism and a value at one indicates an additive effect of the compounds being tested [57].

### 4.5. DNA and RNA Isolation

Cellular DNA from human mammary epithelial cells was isolated after 20 h incubation of a cell lysate with proteinase K followed by extraction using phenol:chloroform:isoamyl alcohol (25:24:1) mixture (Sigma-Aldrich, Poznan, Poland) according to the manufacturer’s protocol. Pure DNA was resuspended in TE buffer and stored at −20 °C. Total RNA from cells was isolated using TRIZOL (Invitrogen, Life Technologies, Carlsbad, CA, USA) according to the manufacturer’s protocol. Isolated RNA was resuspended in water containing 1% DEPC (ribonuclease inhibitor) and stored at −70 °C.

### 4.6. Methylation-Sensitive Restriction Analysis (MSRA)

Methylation status of *RARB* and *PTEN* proximal promoter regions in MCF7, MDA-MB-231, and MCF10A mammary epithelial cells was estimated using methylation-sensitive restriction analysis (MSRA) according to Iwase’s method [63]. Genomic DNA (0.5 μg) isolated from control (unexposed) and exposed cells was incubated at 37 °C overnight with HpaII (20U) restriction enzyme (Fermentas, Vilnius, Lithuania) recognizing non-methylated C↓CGG sequence in *RARB* [22,23] and *PTEN* [24,25] promoter fragments. Specific promoter fragments were chosen for methylation analysis taking into consideration literature data, the analysis of promoter regions using CpGplot software (version r6, London, UK) [64], as well as the analysis of publicly available Illumina 450K data (GSE66695), which indicated the key role of these fragments in regulation of gene transcriptional activity. The fragment of *RARB* promoter includes two retinoic acid response elements (RAREs) and three methylation-sensitive CpG dinucleotide sequences located close to the RAREs [22]. *PTEN* promoter fragment encompasses one HpaII site near the binding sequence for AP-4 (activator protein-4) methylation-sensitive transcription factor [24,25]. A sample without the restriction enzyme and a sample digested with MspI (Fermentas) were incubated in the same conditions, and used as controls of digestion. After incubation, control and digested DNA were amplified using primers listed in Table 2, designed using free online tool for primer design Primer3 [65,66]. The reaction mixture for polymerase chain reaction (PCR) was prepared as described previously [10] and carried out in Tpersonal Thermal Cycler (Biometra, Goettingen, Germany) at 95 °C for 5 min, cycled 30 times for 1 min at 94 °C, 1 min at annealing temperature (Table 2) and 1 min at 70 °C, followed by 10 min extension at 72 °C. The amplified PCR products were fractioned on a 6% polyacrylamide gel, stained with ethidium bromide and visualised under UV illumination. Densitometry analysis of band intensity was performed using the QuantityOne software (Bio-Rad Laboratories Ltd., Watford, UK). Methylation level in each sample was expressed as a percentage of undigested DNA after comparison of band intensities for digested and undigested DNA from the same sample. Our assays did not include promoter methylation analysis of *DNMT1*, *CDKN1A*, and *TP53*. According to literature data, other epigenetic modifications and non-epigenetic mechanisms might be involved in regulation of transcriptional activity of these genes in breast tumors [33,67,68,69].

### 4.7. Quantitative Real-Time PCR (qPCR)

cDNA was synthesized using 2 μg of total RNA, 6 μL of random hexamers, 5 μL of oligo(dT)_15_, and ImProm-II reverse transcriptase (Promega, Mannheim, Germany). The mixture was incubated at 70 °C for 10 min and then reaction with ImProm-II reverse transcriptase (Promega, Mannheim, Germany) was conducted according to the manufacturer’s protocol. The samples were incubated under the following conditions: 5 min at 25 °C, 60 min at 42 °C, and 15 min at 70 °C. cDNA was stored at −20 °C. qPCR was carried out in the Rotor-Gene TG-3000 (Corbett Research, Sydney, Australia). The single reaction mixture contained the following: 2 μL of 10× PCR buffer (100 mM-Tris-HCl, pH 9.0; 500 mM-KCl; 1% Triton X-100), 2 mM MgCl_2_ (Promega, Mannheim, Germany), deoxyribonucleotide triphosphate mix (200 mM each; Promega, Mannheim, Germany), forward and reverse primers (200 nM each; IBB, Warsaw, Poland), 1 μL of 20× EvaGreen fluorescence dye (Biotium, Hayward, CA, USA), two units of Taq polymerase (Promega, Mannheim, Germany), and 1 μL of cDNA in a final volume of 20 μL [3]. The reaction mixture comprised primers listed in Table 3 that were designed using Primer3 (Cambridge, MA, USA) [65,66]. For expression analysis of *RARB* and *PTEN*, as well as *DNMT1*, *CDKN1A*, and *TP53*, the primers were established so that they overlapped splice junction, thereby avoiding the potential amplification of genomic DNA.

After an initial 2 min denaturation step at 94 °C, amplification consisted of fifty cycles was performed under the following conditions: 30 s at 94 °C, 15 s at annealing temperature (Table 3), and 30 s elongation at 72 °C. The relative expression of each tested gene was normalized to the geometric mean of three housekeeping genes, *H3F3A* (H3 histone family 3A), *RPLP0* (60S acidic ribosomal protein P0), and *RPS17* (40S ribosomal protein S17) [3], according to Pfaffl’s method [70].This combination of genes showed the most stable reference in previous reports [3,4,6,10,11,54,71,72].

### 4.8. Statistical Analysis

Statistical analysis of cell viability, apoptosis, MSRA, and qPCR assays was performed using the unpaired *t*-test with two-tailed distribution. Each value represents the mean ± SD of three independent experiments. The results were considered statistically significant when *p* < 0.05.

## Figures and Tables

**Table 1:** Effects of ClF at IC_50_ concentration (640 nM and 50 nM in MCF7 and MDA-MB-231 cells, respectively), EGCG or genistein at 10 µM concentration, as well as ClF (IC_50_) in combination with EGCG or genistein (10 µM) on MCF7 and MDA-MB-231 cell viability ((viable exposed/viable vehicle control)*100%).

**A**	**Cell Viability**			
	**MCF7**		**MDA-MB-231**	
ClF (IC_50_)	53.6 ± 2.5 ***		54.2 ± 2.7 ***	
EGCG 10 µM	87.2 ± 7.6 *		83.3 ± 8.9 *	
Genistein 10 µM	79.7 ± 8.1 *		78.3 ± 13.0 *	
ClF + EGCG	37.1 ± 3.1 ***^^^##		45.2 ± 2.7 ***^^#	
ClF + Genistein	39.9 ± 1.1 ***^^###		44.9 ± 3.6 ***^#	
**B**	**MCF7**		**MDA-MB-231**	
ClF (IC_50_)	+	+	+	+
EGCG 10 µM	+	−	+	−
Genistein 10 µM	−	+	−	+
Average CI	0.8021 ± 0.0458 ***	1.1439 ± 0.0913	0.7825 ± 0.0593 **	1.0808 ± 0.1065

(**A**) All results represent mean ± SD of three independent experiments. Exposure (ClF alone, EGCG alone, genistein alone, ClF + EGCG, or ClF + Genistein) versus vehicle control, *** *p* < 0.001, ** *p* < 0.01, * *p* < 0.05. EGCG or genistein alone versus combinatorial exposure (ClF + EGCG or ClF + Genistein), ^^^ *p* < 0.001, ^^ *p* < 0.01, ^ *p* < 0.05. ClF alone versus combinatorial exposure (ClF + EGCG or ClF + Genistein), ### *p* < 0.001, ## *p* < 0.01, # *p* < 0.05. (**B**) CompuSyn data of trypan blue exclusion test values [57] (cell growth inhibition, (viable exposed/viable vehicle control)) indicate synergistic (CI < 1) effect of ClF + EGCG and nearly additive (CI = 1) effect of ClF + Genistein in both MCF7 and MDA-MB-231 breast cancer cells. CI, combination index.

**Table 2:** Primer sequences, annealing temperature, and polymerase chain reaction (PCR) product size used for methylation-sensitive restriction analysis (MSRA).

Gene	Amplicon Length (bp)	Sequence of Primers (5′ → 3′) F—Forward; R—Reverse	Annealing Temperature (°C)	UCSC RefSeq Gene Accession NM (Human GRCh37/hg19 Assembly)
*RARB*	295	F: CTCGCTGCCTGCCTCTCTGG	58.4	NM_016152
		R: GCGTTCTCGGCATCCCAGTC		chr:3
*PTEN*	214	F: CAGCCGTTCGGAGGATTATTC	61.1	NM_000314
		R: GGGCTTCTTCTGCAGGATGG		chr:10

**Table 3:** Primer sequences, annealing temperature, and PCR product size used for quantitative real-time PCR (qPCR).

Gene	Amplicon Length (bp)	Sequence of Primers (5′ → 3′) F—Forward; R—Reverse	Annealing Temperature (°C)
*RARB*	92	F: TTCAAGCAAGCCTCACATGTTTCCA	58.4
		R: AGGTAATTACACGCTCTGCACCTTTAG	
*PTEN*	330	F: CGAACTGGTGTAATGATATGT	50.0
		R: CATGAACTTGTCTTCCCGT	
*DNMT1*	100	F: ACCGCCCCTGGCCAAAGCCATTG	60.0
		R: AGCAGCTTCCTCCTCCTTTATTTTAGCTGAG	
*CDKN1A*	103	F: GCTCAGGGGAGCAGGCTGAAG	60.0
		R: CGGCGTTTGGAGTGGTAGAAATCTGT	
*TP53*	120	F: TAACAGTTCCTGCATGGGCGGC	64.0
		R: GGACAGGCACAAACACGCACC	
*H3F3A*	76	F: AGGACTTTAAAACAGATCTGCGCTTCCAGAG	65.0
		R: ACCAGATAGGCCTCACTTGCCTCCTGC	
*RPLP0*	69	F: ACGGATTACACCTTCCCACTTGCTGAAAAGGTC	65.0
		R: AGCCACAAAGGCAGATGGATCAGCCAAG	
*RPS17*	87	F: AAGCGCGTGTGCGAGGAGATCG	64.0
		R: TCGCTTCATCAGATGCGTGACATAACCTG	

## Abbreviations

ClF	Clofarabine, 2-chloro-2′-fluoro-2′-deoxyarabinosylade
DAC	Decitabine, 5-aza-2′-deoxycytidine
EGCG	(−)-Epigallocatechin gallate
